# The Activity of *Solanum tuberosum* Leaf Extract and Chaconine in the Gut of *Tenebrio molitor* Larvae

**DOI:** 10.3390/toxins18040157

**Published:** 2026-03-26

**Authors:** Malgorzata Slocinska, Justyna Mirek, Zbigniew Adamski, Jan Lubawy

**Affiliations:** 1 Department of Animal Physiology and Developmental Biology, Faculty of Biology, Adam Mickiewicz University in Poznan, 61-614 Poznan, Poland; justyna.mirek@gliwice.nio.gov.pl (J.M.); ed@amu.edu.pl (Z.A.); j.lubawy@amu.edu.pl (J.L.); 2 Electron and Confocal Microscope Laboratory, Faculty of Biology, Adam Mickiewicz University in Poznan, 61-614 Poznan, Poland

**Keywords:** glycoalkaloids, insect, mealworm, digestive enzymes activity, gut contractility

## Abstract

Steroidal glycoalkaloids (GAs) are key plant defense compounds, yet their effects on insect gut physiology are not fully understood. We investigated how purified α-chaconine and *Solanum tuberosum* leaf extract influence the gut function and growth of the mealworm *Tenebrio molitor*. Larvae were exposed to sublethal doses of GAs, and gut contractility, midgut digestive enzyme activity and body weight were analysed over time. Both α-chaconine and potato extract caused a rapid decrease in digestive enzyme activity 2 h after exposure, followed by a clear increase above control levels after 24 h, indicating a time-dependent compensatory response of the digestive system. Gut contractility was significantly enhanced in treated larvae, and larvae exposed to both treatments exhibited a body weight loss over 72 h. These results show that potato glycoalkaloids strongly modulate the gut physiology of *T. molitor* while allowing continued growth, highlighting both the plasticity of insect digestive responses and the need to consider sublethal, gut-centered effects when evaluating glycoalkaloids as candidates for bioinsecticidal agents.

## 1. Introduction

Glycoalkaloids (GAs) are secondary metabolites of the family *Solanaceae* and are commonly found in economically important and widely harvested crops such as potato (*Solanum tuberosum* L.), tomato (*Solanum lycopersicum* L.) and eggplant (*Solanum melongena* L.). Historically, “solanine” was shown in the 1950s to comprise two major glycoalkaloids, α-solanine and α-chaconine, that share the steroidal alkaloid aglycone solanidine, with subsequent work (1960s–1970s) clarifying the fundamental aspects of GAs chemistry [1,2,3]. The two major GAs in cultivated potato, α-solanine and α-chaconine, are glycosylated derivatives of the aglycone, solanidine, and account for more than 90% of the total glycoalkaloid content [4]. α-Solanine bears the trisaccharide solatriose, whereas α-chaconine bears chacotriose, features that influence membrane interactions and bioactivity [5,6]. They accumulate in all plant organs, including tubers, sprouts, flowers, leaves and stems, with particularly high concentrations found in photosynthetic and reproductive tissues [7,8]. GAs levels vary with genotype and are modulated by light exposure, wounding/sprouting, and storage/temperature conditions, which are important for both plant defense and food safety [8,9,10,11].

Potato glycoalkaloids contribute significantly to plant resistance against fungi, bacteria, nematodes and other herbivorous animals [12,13] and therefore act as natural pesticides within plants [14]. On the other hand, when present at high levels in edible tissues, α-solanine and α-chaconine are problematic for food and feed safety because of their toxicity to vertebrates [15,16]. Their biological activity is attributed mainly to their ability to interact with membrane sterols and disrupt cellular membranes as well as to inhibit acetylcholinesterase activity, which can affect neuromuscular and autonomic regulation [17,18,19]. Beyond direct toxicity, GAs can induce a wide spectrum of physiological effects, including oxidative stress [12,17,20], perturbation of energy metabolism [21,22] and disruption of ion and nutrient homeostasis [5,23,24]. In insects, sterol-dependent membrane disruption in midgut epithelia, potential effects on digestive enzymes and ion balance, and reported synergy between α-solanine and α-chaconine provide a mechanistic basis for insecticidal activity [25,26]. Potato leaves, which are byproducts of potato cultivation, represent a rich source of α-solanine and α-chaconine and have been proposed to be composed of plant-derived preparations with insecticidal activity. They could be used in Integrated Pest Management (IPM) whose assumptions are based on the use of a mixture of various methods for plant protection, including prevention, substances of natural origin, or mechanical control [27].

The combination of potent bioactivity and plant origin has driven interest in GAs and GA-rich extracts as potential botanical insecticides, provided that their selectivity and mechanisms of action in target insects are sufficiently understood. Studies have shown that extracts obtained from Solanaceae plants exhibit antibacterial, antifungal and zoocidal activity for review see: [13,17,28]. Insect responses to Solanaceae secondary metabolites frequently involve changes in midgut physiology, including alterations in the epithelial structure and ultrastructure of fat body tissue [29,30], as well as ovicidal and repellent activity [31,32]. Moreover, they impact reproduction and development and induce oxidative stress [20,33]. However, detailed mechanisms of their action in insects are still lacking.

In terms of Solanaceae glycoalkaloids, several recent works using the yellow mealworm *Tenebrio molitor* have demonstrated that exposure to pure GAs or Solanaceae extracts affects hemolymph nutrient profiles, lipid homeostasis, β-oxidation enzyme activity and the antioxidant system in the fat body and other tissues, indicating profound interference with energy management and redox balance [20,21,34,35]. Recently, Solanaceae secondary metabolites were shown to modulate the immune system activity of *T. molitor* [36] and induce changes in the retrocerebral complexes of Tenebrionidae beetles [37]. *T. molitor*, which belongs to the order Coleoptera, is a cosmopolitan stored-product pest that feeds on a variety of cereal-based products and is among the most important model organisms in insect physiology and toxicology [38,39]. Moreover, it is being increasingly reared on an industrial scale as a source of proteins and lipids for food and feed, which makes it a species of considerable economic and ecological relevance [40,41]. Given the high GAs content in *S. tuberosum* leaves and the growing interest in valorizing agricultural byproducts as sources of bioactive compounds, it is important to characterize precisely how complex leaf extracts and individual glycoalkaloids affect the biochemical and physiological processes of nontarget and target insects.

The insect gut is the primary site of nutrient digestion and absorption and constitutes the first physiological barrier encountered by dietary chemicals and microbial metabolites [42,43]. However, relatively little is known about how potato-derived glycoalkaloids or potato leaf extracts influence the gut itself, despite it is being a critical target for dietary toxins and a key determinant of insect performance and survival. Differences between a crude extract and a single purified GA may reveal synergistic or antagonistic interactions between the metabolites included in the extract, which could either enhance or mitigate its toxicity. Moreover, understanding the midgut-specific responses of *T. molitor* larvae to potato glycoalkaloids is relevant both for evaluating their potential as botanical insecticides and for assessing possible risks when mealworms are reared on plant-based side streams containing GAs. Therefore, the present study aimed to determine the activity of *S. tuberosum* leaf extract and its major secondary metabolite, α-chaconine, on the gut of *Tenebrio molitor* larvae. We specifically sought to compare the effects of complex leaf extract with those of a single compound on selected physiological parameters of the *T. molitor* larval gut, such as motility, digestive enzyme activity, and body weight, to clarify the effects of glycoalkaloids and the gut response to α-chaconine and *S. tuberosum* extract.

## 2. Results

### 2.1. Effects of α-Chaconine and S. tuberosum Extract on Hindgut Motility

**Figure 1:**
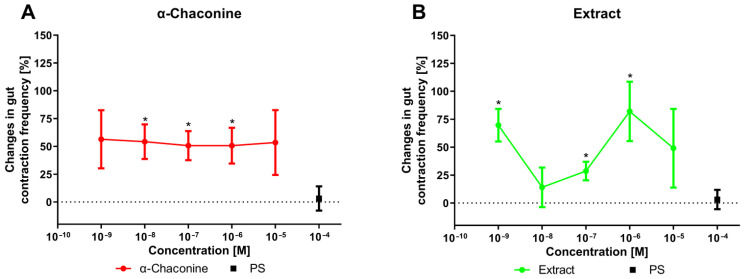
Changes in in vitro hindgut contractile activity of *T. molitor* after application with α-Chaconine (**A**) and *S. tuberosum* extract (**B**) compared with the control at concentrations ranging from 10^−9^ to 10^−5^ M. Means ± SEM are given for *n* = 10. Significant differences from the control (physiological saline: PS) were determined using Student’s *t* test and are indicated by * *p* ≤ 0.05. Scatter dot plots are presented in Figure S1.

Under in vitro conditions, the hindgut of *T. molitor* exhibits irregular endogenous activity. Under constant perfusion with physiological saline (PS), the average frequency of hindgut contractions was 5.04 ± 3.53 contractions per minute. The application of an additional 10 μL of PS did not cause any significant changes in contractile activity. However, when tested in the concentration range of 10^−9^ to 10^−5^ M, alkaloids induced myostimulatory effects (positive chronotropic) (Figure 1). α-Chaconine exerted a statistically significant myostimulatory effect at concentrations between 10^−8^ and 10^−6^ M (10^−8^ M: *p* = 0.0176; 10^−7^ M: *p* = 0.0135; 10^−6^ M: *p* = 0.0289) (Figure 1A). The effect remained relatively constant at approximately 50% (ranging from 56% to 50.7%). Compared with α-chaconine alone, potato extract also increased the contraction frequency, although the response was more irregular (Figure 1B). Statistically significant changes were observed at concentrations of 10^−9^, 10^−7^, and 10^−6^ M, corresponding to increases of 69.7% (*p* = 0.0012), 28.7% (*p* = 0.0481), and 82.1% (*p* = 0.0481), respectively.

### 2.2. Effects of α-Chaconine and S. tuberosum Extract on the Weight Loss of T. molitor

**Figure 2:**
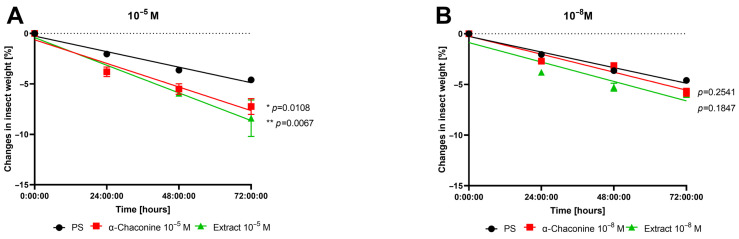
Weight loss of *T. molitor* over 72 h after the application of α-chaconine (red) and *S. tuberosum* extract (green) at concentrations of 10^−5^ (**A**) and 10^−8^ M (**B**). The values are presented as the mean ± SEM from three independent replicates (*N* = 3), each containing 20 individuals (*n* = 20). Linear regression analysis was used to assess differences among the curves. Significant differences between treatments and control regression lines (physiological saline: PS) were evaluated using an F-test equivalent to analysis of covariance (ANCOVA) and are indicated by * *p* ≤ 0.05 or ** *p* ≤ 0.01. Scatter dot plots are presented in Figure S2.

Considering changes in gut motility, we next measured larval weight over three days. Linear regression analysis was used to assess changes in insect mass over time in control insects (PS) and those injected with α-chaconine or *S. tuberosum* extract at concentrations of 10^−5^ M and 10^−8^ M (Figure 2). Both pure glycoalkaloid and potato extract significantly decreased the rate of mass loss at 10^−5^ M, with the extract having the strongest effect. For PS- versus α-chaconine-injected insects, the slopes differed significantly (F = 7.904, *p* = 0.0108), indicating that compared with the controls, α-chaconine accelerated mass loss. Similarly, the slopes of the PS-injected and potato extract-injected insects significantly differed (F = 9.154; *p* = 0.0067). Conversely, at the lower concentration (10^−8^ M), neither the slopes of the PS-injected insects nor the α-chaconine-injected insects nor the slopes of the PS-treated and extract-treated insects significantly differed (F = 1.379, *p* = 0.2541 and F = 1.887, *p* = 0.1847, respectively).

#### Correlation Analysis

**Figure 3:**
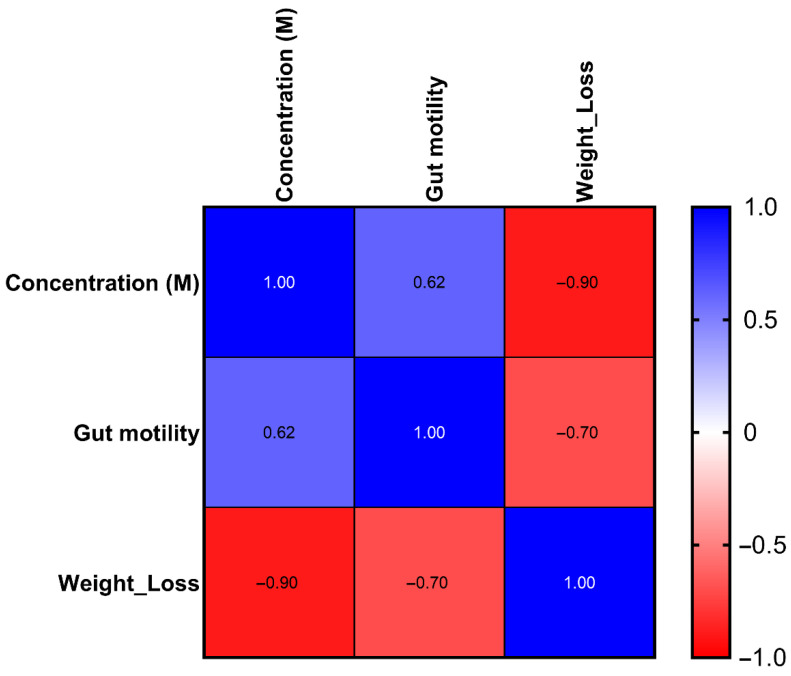
Correlation matrix illustrating relationships among glycoalkaloid concentration, gut motility, and weight loss. The color intensity represents the strength and direction of the Pearson correlation coefficient (r), which ranged from −1 (strongly negative correlation, red) to +1 (strongly positive correlation, blue). Annotated values indicate correlation strength. The raw data used to generate the matrix are presented in Figure S3.

Next, we constructed a correlation matrix to reveal associations between the concentrations of the tested compounds, gut motility and weight loss (Figure 3). The concentration was positively correlated with gut motility (r = 0.616), indicating that higher alkaloid concentrations were associated with increased intestinal activity. In contrast, concentration strongly negatively correlated with weight loss (r = −0.896), suggesting that higher concentrations were linked to greater body mass reduction. Gut motility was also negatively correlated with weight loss (r = −0.705), indicating that individuals with higher motility tended to lose more weight. These findings highlight the physiological relationships among alkaloid exposure, gastrointestinal function, and weight dynamics.

### 2.3. Effects of α-Chaconine and S. tuberosum Extract on Digestive Enzyme Activity

**Figure 4:**
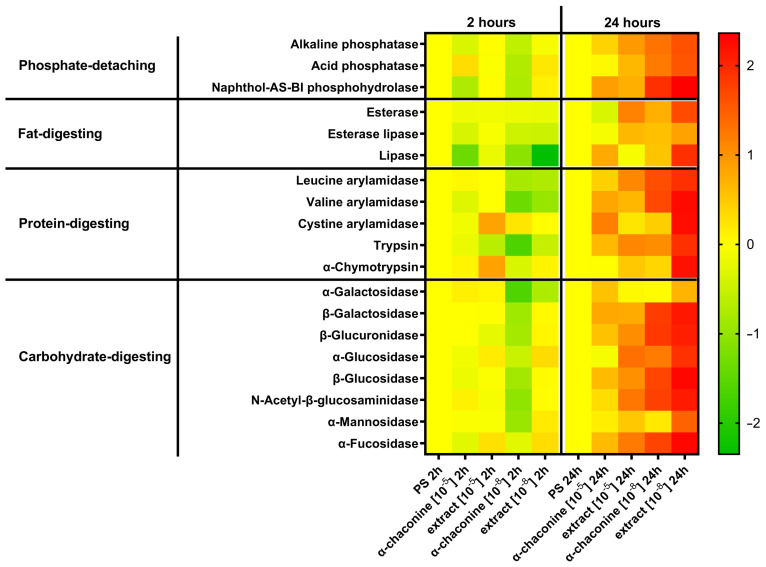
Changes in the activity of digestive enzymes 2 and 24 h after the application of α-chaconine, *S. tuberosum* extract and physiological saline (PS) at 10^−8^ and 10^−5^ M concentrations. Changes in the compound levels are marked in yellow (no change), green (decrease), and red (increase). The data are presented as log2FC values, with the scale indicating higher (value >  0) or lower (value <  0) metabolite levels than those of the control (value =  0). The statistics for the figure are presented in Figure S4.

The use of API-zym allowed us to measure the activity of 19 enzymes in the isolated guts of *T. molitor*, namely: Alkaline phosphatase, Esterase, Esterase lipase, Lipase, Leucine arylamidase, Valine arylamidase, Cystine arylamidase, Trypsin, α-Chymotrypsin, Acid phosphatase, Naphthol-AS-BI phosphohydrolase, α-Galactosidase, β-Galactosidase, β-Glucuronidase, α-Glucosidase, β-Glucosidase, N-Acetyl-β-glucosaminidase, α-Mannosidase, and α-Fucosidase. A Three-Way ANOVA was conducted to examine the effects of dose (10^−5^ M and 10^−8^ M) and treatment (α-chaconine and potato extract) on the activity of all the enzymes after both 2 h of treatment and 24 h of treatment (Figure 4). In terms of all the enzymes analysed, compared with the PS and chaconine treatments, the 10^−8^ M potato extract treatment consistently resulted in the highest activity at 24 h, as well as earlier time points. For acid phosphatase activity, significant main effects of time (*p* = 0.0083) and dose (*p* = 0.0418) were detected, along with a time × dose interaction (*p* = 0.0088). Post hoc analysis confirmed that the extract concentration at 10^−8^ M after 24 h was significantly greater than those of PS and α-chaconine (*p* = 0.0152–0.0214) and earlier extract exposures (*p* = 0.0400). Naphthol-AS-BI phosphohydrolase exhibited significant effects on treatment (*p* = 0.0415), time (*p* = 0.0034), and dose (*p* = 0.0116), with interactions for treatment × time (*p* = 0.0202) and time × dose (*p* = 0.0143). Tukey’s test revealed that the extract at 10^−8^ M after 24 h exceeded PS and α-chaconine (*p* = 0.0012–0.0024) and earlier extract time points (*p* = 0.0110–0.0290). Among glycosidases, α-Galactosidase, β-Galactosidase, β-Glucuronidase, α-Glucosidase, and β-Glucosidase showed significant main effects on treatment, time, and dose (*p* < 0.05), with interactions primarily involving treatment × time and time × dose. In all the cases, the extract concentration at 10^−8^ M after 24 h was markedly greater than those of PS and α-chaconine (*p* values ranging from 0.0001 to 0.0491) and earlier extract exposures. For N-Acetyl-β-glucosaminidase, treatment (*p* = 0.0216), time (*p* = 0.0011), and dose (*p* = 0.0242) were significant, as were treatment × time and time × dose interactions. Post hoc tests confirmed that the extract concentration at 10^−8^ M after 24 h was significantly greater than those of PS and α-chaconine (*p* = 0.0005–0.0077) and earlier extract exposures (*p* = 0.0047–0.0059). α-Mannosidase showed no significant main effects (*p* > 0.05), but treatment × dose (*p* = 0.0173) and time × dose (*p* = 0.0366) interactions were detected. The concentration of the extract at 10^−8^ M after 24 h was greater than that of PS and chaconine in the selected comparisons (*p* = 0.0100–0.0291). Finally, α-Fucosidase demonstrated significant main effects of treatment (*p* = 0.0140), time (*p* = 0.0016), and dose (*p* = 0.0121), with interactions for treatment × time (*p* = 0.0291) and time × dose (*p* = 0.0131). Post hoc analysis revealed that the extract at 10^−8^ M after 24 h was significantly higher than PS and chaconine (*p* = 0.0005–0.0151) and earlier extract exposures (*p* = 0.0045–0.0049).

Overall, the most consistent pattern across enzymes was a pronounced increase in activity following 24 h of exposure to *S. tuberosum* extract at 10^−8^ M, indicating a stronger stimulatory effect than that of the PS and α-chaconine treatments.

## 3. Discussion

Solanaceae glycoalkaloids are well established as multitarget defense compounds that affect the survival, growth, metabolism and reproduction of herbivorous insects [13,44]. In the model insect, *T. molitor* beetle, *Solanum nigrum* fruit extract and pure glycoalkaloids cause sublethal changes in lipid, glycogen, and protein content, ultrastructural damage in the fat body and the midgut and altered contractility of visceral muscles [30]. Recent studies have shown that in *T. molitor* beetle, solanine, α-chaconine, tomatine and tomato extracts disturb lipid metabolism and reduce the activity of 3-hydroxyacyl-CoA dehydrogenase (HADH) in the fat body, disrupt hemolymph nutrient homeostasis and modulate the antioxidant system [21,34]. Insect hemolymph is a key mediator of nutritional and immunological homeostasis in insects, and the fat body plays a crucial role in insect intermediary metabolism, indicating that GAs trigger systemic metabolic and oxidative stress responses in the insect body.

In our study, we report the dynamic response of the digestive system, reflecting changes in the enzyme activity, gut motility and body weight of *T. molitor* beetles under GAs treatment. The application of α-chaconine and *S. tuberosum* extract to *T. molitor* larvae produced concentration- and time-dependent responses of the digestive system: (i) a rapid decrease in gut digestive enzyme activity 2 h after treatment, followed by (ii) a marked increase in the same enzymes 24 h later, (iii) a sustained increase in gut contractility, and (iv) a reduction in larval body mass. The rapid reduction in digestive enzyme activity 2 h after glycoalkaloid or extract application is consistent with an acute “shock” phase in which digestive processes are temporarily suppressed. Plant secondary metabolites, including alkaloids, are known to inhibit insect digestive enzymes directly or indirectly, for example, by binding to enzyme proteins, changing the luminal pH, or damaging midgut epithelial cells [45,46]. In line with these observations, antifeedant activity of these compounds has been reported [47]. In addition, steroidal glycoalkaloids disrupt biological membranes and inhibit acetylcholinesterase, altering cholinergic signalling in neuromuscular and secretory pathways [5,48]. In this study, after the application of α-chaconine and *S. tuberosum* extract, we observed a similar pronounced increase in gut contractility compared with that in the control group. The augmented intestinal peristalsis following glycoalkaloids administration suggests that these compounds disturb the physiological regulation of gastrointestinal motility. Insects control gut motility primarily through peptide and aminergic signalling (e.g., proctolin, allatostatins, and octopamine), with glutamate acting as a common fast transmitter at visceral neuromuscular junctions [42,49]. Solanaceae glycoalkaloids such as α-chaconine are known AChE inhibitors and potent membrane-active compounds. Therefore, the observed changes in gut contractility are likely to arise from a combination of (i) disruption of neuromuscular and epithelial membranes and (ii) indirect modulation of neuromodulatory circuits, with AChE inhibition contributing but probably not representing the sole mechanism. Winkiel et al. [35] reported that chaconine and solanine were detected in the *T. molitor* gut after injection for 24 h, which indicates that GAs transported from the haemolymph or by Malpighian tubules may evoke a direct toxic effect and alter gut physiology without entering the insect body with food.

Modulation of the activity of the hindgut and other visceral muscles by *S. nigrum* extract and pure glycoalkaloids (solanine and solasomargine) has been demonstrated in *T. molitor*, where increased contraction frequency and amplitude were proposed to contribute to impaired development, food intake and reproduction [30]. Enhanced peristalsis, together with reduced digestive enzymes’ activity, presumably accelerates food passage through the gut while simultaneously lowering the capacity to chemically process the ingesta. 24 h after treatment, digestive enzyme activity exceeded control levels. Such delayed upregulation may indicate an adaptive or compensatory response to the earlier digestive impairment. Herbivorous insects commonly adjust both digestive and detoxification enzymes when exposed to allelochemicals, increasing the production of proteases, carbohydrases and xenobiotic-metabolizing enzymes to maintain nutrient acquisition and limit toxin damage [45,50].

In our case, the biphasic pattern (early inhibition, later overcompensation) may reflect either direct inhibitory and membrane-disrupting actions of glycoalkaloids dominating in the first hours or transcriptional and translational upregulation of digestive enzymes as larvae attempt to restore digestive capacity under continued exposure. This interpretation parallels the time-dependent impact of glycoalkaloids on energy metabolism observed in *T. molitor*, reflected in fluctuations in HADH activity and lipid levels at both 2 h and 24 h after glycoalkaloid injection, with effects depending on compound, concentration and tissue [21]. Thus, similar mechanisms may apply to the digestive system, where initial functional impairment is followed by metabolic reprogramming toward increased digestive and detoxification capacity. Moreover, glycoalkaloids modulate the antioxidant system in *T. molitor*, altering the activities of key enzymes such as superoxide dismutase and catalase [20]. Because oxidative stress responses and detoxification are energetically expensive, their activation may enhance digestive efficiency, which could help explain the elevated digestive enzyme activities at 24 h.

A statistically significant increase in the frequency of intestinal contractions was observed in the α-chaconine- and *S. tuberosum* extract-treated groups, which correlated with a decrease in larval weight compared with that in the control groups. In our assays, the *T. molitor* larvae had constant access to food for the entire 72 h of exposure after glycoalkaloids were delivered by hemocoelic injection; therefore, short-term mass changes are likely to be a consequence of gut increased motility and accelerated passage of food through intestine induced by GAs, leading to negative energy balance. Alterations in gut motor function play a central role in the antinutritional effects of glycoalkaloids; modulation of hindgut contractility by Solanaceae glycoalkaloids and extracts has been proposed as a key component of their sublethal toxicity in *T. molitor*, potentially affecting food intake and transit [30]. Faster or dysregulated peristalsis could shorten retention time in the midgut, limiting the window for enzymatic digestion and nutrient absorption even when enzyme levels rebound. This interpretation is consistent with the general principles of insect digestion, where transit/retention time is a primary determinant of assimilation efficiency [51].

Thus, even if the weight of the larvae did not change significantly, some tendencies were observed, likely because of a combination of reduced digestive efficiency, disturbed energy metabolism, or/and increased metabolic costs of defense. This finding is supported by the abovementioned findings of reduced HADAH activity and lipid levels, indicating that the increased metabolic cost likely accelerates weight loss. Our observation of weight loss by insects within 72 h after glycoalkaloid application may indicate that *T. molitor* larvae are pushed into a negative energy balance when digestive impairment, hypermotility and metabolic stress cooccur. Because we did not quantify food intake or frass and relied on wet mass, we cannot fully partition short-term mass decreases between fluid/contents loss and tissue catabolism; incorporating frass (dry-mass) output and larval dry-mass endpoints in future assays would resolve this [52]. The extended exposure of *T. molitor* larvae to *S. nigrum* extract and pure glycoalkaloids, in consequence, may alter the contents of glycogen, lipids and proteins in the midgut and fat body even without causing acute mortality [30]. Notably, our 72 h mass decrease contrasts with that reported by Spochacz et al. [30], who delivered *S. nigrum* glycoalkaloids with food and emphasized sublethal physiological changes with slight weight gain; the discrepancy likely reflects differences in glycoalkaloid source/profile (*S. tuberosum* vs. *S. nigrum*), exposure route (injection vs. diet), and dose–related factors known to shift potency, with α-chaconine often exhibiting stronger bioactivity than α-solanine depending on context. Similarly, nutritional studies conducted by Nenaah [47] revealed a significant reduction in the growth rate, food consumption and food utilization by adult *Trogoderma granarium* beetles treated with potato glycoalkaloids. This broader antinutritional pattern in stored-product beetles supports the view that sublethal glycoalkaloid exposure can rapidly impair performance even in the absence of mortality.

In our study, both the α-chaconine and the *S. tuberosum* extract affected the measured parameters in a similar way, except for digestive enzymes activity, where greater efficacy was observed for the potato extract. Compared with α-chaconine alone, other glycoalkaloids and secondary metabolites identified in *S. tuberosum* extract [33] may modulate enzyme responses, potentially enhancing stress and weight loss. A stronger response of the hindgut muscle to extracts than to single compounds has been described for *S. nigrum* fruit extract, which contains a mixture of glycoalkaloids and produces physiological effects that differ from those of pure solasonine or solamargine, indicating additive or synergistic interactions among components [30].

## 4. Conclusions

In summary, the effects observed in this study indicate that Solanaceae glycoalkaloids act as broad modulators of insect digestive physiology, shifting the balance between nutrient acquisition, digestive system metabolism, detoxification, and energy expenditure in a way that ultimately impairs growth. From an applied perspective, the combination of early digestive inhibition, later metabolic dysregulation, and progressive weight loss makes glycoalkaloids and Solanaceae extracts promising candidates for use as bioinsecticides that impair pest performance even when mortality is low. However, further studies involving the feeding of insects with plants containing GAs or the preparation of an artificial diet supplemented with GAs are needed. Although the administration of GAs by injection allows one to determine the precise concentration of applied GAs, under natural conditions, GAs enter the insect body with food, and the gut is the first place in which these toxic compounds are in contact. Such a study allows for the interpretation of studies related to the effects of potato GAs on the animal digestive system and gut physiology and for the estimation of their potential utility in plant protection.

## 5. Materials and Methods

### 5.1. Insects

Larvae of *T. molitor* were obtained from a culture maintained in the Department of Animal Physiology and Developmental Biology, Poland, under constant temperature (26 ± 0.5 °C), humidity (65 ± 5%) and a 12:12 h light:dark cycle. The food consisted of oat flakes and fresh carrots as described previously [53]. Because *T. molitor* insects do not have a fixed number of larval instars, only feeding larvae from the 15th to 16th instar of approximately 100–150 mg of weight were selected for the experiments.

### 5.2. Chemical Standard and Plant Extract

Pure α-chaconine was obtained from Lab Service Analytica. The standard was dissolved in physiological saline (PS) for beetles (274 mM/L NaCl, 19 mmol/L KCl, and 9 mmol/L CaCl_2_) to obtain a 10^−3^ M concentration. The samples were stored at −20 °C. Immediately before use, the dilutions of the tested compounds were prepared to the desired concentration. To obtain extracts, freshly collected potato leaves were prepared as previously described by Ventrella et al. [33]. The extract of *S. tuberosum* leaves contained mainly α-chaconine and α-solanine, together with other minor GAs. The samples were dissolved in PS containing 0.1% acetic acid to obtain a 10^−3^ M concentration for their main component metabolite α-chaconine and stored at −20 °C before use. Immediately before use, the dilutions of test compounds were prepared to the desired concentration. The tested solutions of α-chaconine and *S. tuberosum* extract were injected into the insect in the form in a volume of 2 uL at two concentrations: 10^−5^ and 10^−8^ M. These concentrations were selected on the basis of the literature and our previous studies, indicating that they induce different metabolic and developmental disorders [54].

### 5.3. Analysis of Insect Weight

Larvae of *T. molitor* were divided into 5 experimental groups (each group containing a minimum of 20 individuals), a control group (injected with PS), insects injected with α-chaconine at either 10^−8^ or 10^−5^ M, and insects injected with extract at concentrations 10^−8^ and 10^−5^ M, and placed in plastic containers with constant access to food. The weight of the larvae was monitored for 72 h. The results were calculated as the dynamic changes in the average weight of the tested larvae at the following measuring points, after 24 h, 48 h, and 72 h, compared to the beginning of the experiment (point 0). Each experiment was repeated in triplicate.

### 5.4. Determination of Midgut Enzymes Activity

Larvae, 100–150 mg in weight were selected for experiments 2 h after and 24 h after glycoalkaloid injection at 10^−8^ and 10^−5^ M, respectively. Dissected larvae digestive tracts from 20 specimens from each experimental group were transferred to Ringer solution at 4 °C and homogenized with 10 strokes. Afterward, the sample was centrifuged for 10 min (3000× *g*) at 4 °C, and the supernatant was used for the analysis of enzyme activity. For the rapid detection of qualitative enzyme activity in samples from the whole midgut homogenate, the commercially available ApiZYMtest (bioMe’rieux Inc., Warsaw, Poland) was used. Measurements according to the manufacturer’s instructions were performed in samples (65 mL) containing approximately 200 mg of protein per milliliter of homogenate. After 4 h of incubation, the developed color was compared in each test cupule with specific enzyme activity. To examine the differences in the enzymes’ activity between the control and experimental samples, pictures of the tests were taken and subsequently analysed with the ImageJ2x program. The number of pixels of the selected surface area, which was the same for each sample, was calculated. Enzyme activity was calculated in pixel units per mg of protein. The results were calculated as the mean pixel value measured at the center of each sample using ImageJ software (version 2) and are presented as the percent change in digestive enzyme activity compared with that of the individuals injected with physiological saline.

### 5.5. Videomicroscopy Analysis of Hindgut Contractility

The effects of α-chaconine and *S. tuberosum* extract on beetle hindgut contractile activity were evaluated using video microscopy coupled with computer-based data acquisition and analysis, as described by Lubawy, et al. [55]. Isolated hindguts were placed in a perfusion well (100 μL) prepared in a 5 cm Petri dish coated with Sylgard and filled with PS. The chamber was mounted horizontally on the stage of an Olympus SZX12 stereomicroscope equipped with an Olympus SC30 camera (Olympus Poland, Warsaw, Poland). Preparations were continuously superfused with fresh PS at a flow rate of 140 μL min^−1^ and stabilized for 10 min prior to recording. Each video was recorded for 2 min; test compounds were applied after 30 s using a Hamilton syringe (10 μL) via the application port in the concentration range of 10^−9^ to 10^−5^ M. Movements at the hindgut edge were traced using AnTracker software (ver. 3.0,PRO MIKRO, Wroclaw, Poland). Myograms were generated by overlaying a point on the binarized and thresholded video image to follow the black/white boundary of the hindgut. Changes in the boundary position between frames were plotted, with each peak corresponding to a single contraction. Contractile activity is expressed as the percentage change relative to the control contraction frequency. Hindguts isolated from ten individuals were tested for each concentration and control. As a positive control, proctolin, a potent myostimulatory insect neuropeptide, was used at a concentration of 10^−5^ M.

### 5.6. Statistical Analysis

To calculate significant differences between samples, GraphPad Prism software ver. 9 (GraphPad Software, San Diego, CA, USA) was used. In the first step, we checked for the normality of the distribution using the Shapiro–Wilk test. For weight loss analysis, linear regression was performed for each group, and differences between treatments and control regression lines were evaluated using an F-test equivalent to analysis of covariance (ANCOVA), as implemented in the software. A correlation matrix of gut motility, concentration and weight loss was constructed using Pearson correlation coefficients. Enzyme activity data were analysed using three-way analysis of variance (ANOVA) with treatment (PS, α-chaconine, and *S. tuberosum* extract), dose (10^−5^ M and 10^−8^ M), and time (2 h and 24 h) as fixed factors. For each enzyme, the percentage of total variation explained by each factor and interaction was calculated. When significant main effects or interactions were detected (*p* ≤ 0.05), Tukey’s multiple comparisons test was applied for post hoc analysis to identify pairwise differences among treatment, dose, and time combinations (for detailed statistics, see Figure S4). All the data are expressed as the mean values ± SD, with the number of replicates (n) specified. Differences were considered statistically significant at the following levels: *p* ≤ 0.05 (*), *p* ≤ 0.01 (**), *p* ≤ 0.001 (***) or *p* ≤ 0.0001 (****).

## Data Availability

The original contributions presented in this study are included in the article/Supplementary Materials. Further inquiries can be directed to the corresponding author.

## Supplementary Materials

The following supporting information can be downloaded at https://www.mdpi.com/article/10.3390/toxins18040157/s1; Figure S1: Scatter dot plots of graphs presented in Figure 1 presenting changes in hindgut contractile activity of *T. molitor* after an application of α-Chaconine (A) and *S. tuberosum* extract (B) compared to the control at concentrations range from 10^−9^ to 10^−5^ M.; Figure S2: Scatter dot plots of graphs presented in Figure 2 presenting weight loss of *T. molitor* over 72 h’ time after application of α-chaconine (red) and *S. tuberosum* extract (green) at concentrations of 10^−5^ (A) and 10^−8^ M (B).; Figure S3: Raw data used to generate correlation matrix presented in Figure 3; Figure S4: Changes in digestive enzyme activity at 2 h and 24 h after injection of α-chaconine, *S. tuberosum* extract, or physiological saline (PS) at concentrations of 10^−5^ M and 10^−8^ M.
