# Supplementary material for: The Activity of Solanum tuberosum Leaf Extract and Chaconine in the Gut of Tenebrio molitor Larvae
Source: Toxins (Basel). 2026 Mar 26;18(4):157. doi: 10.3390/toxins18040157 (PMC13119814; doi:10.3390/toxins18040157)
Supplement: Supplementary file 1 [file toxins-18-00157-s001.zip › toxins-4155314-SI.pdf]

Supplementary Information for:

The Activity of *Solanum tuberosum* Leaf Extract and Chaconine in the Gut of *Tenebrio molitor* Larvae

Malgorzata Slocinska, Justyna Mirek, Zbigniew Adamski and Jan Lubawy

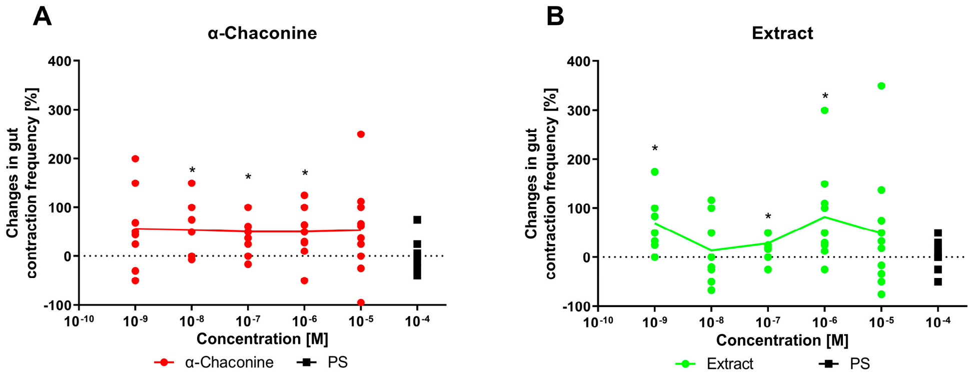

Figure S1. Scatter dot plots of graphs presented in Figure 1 presenting changes in hindgut contractile activity of *T. molitor* after an application of α-Chaconine (A) and *S. tuberosum* extract (B) compared to the control at concentrations range from 10^{-9} to 10^{-5} M. Means ± SEM are given for n = 10. Significant differences from the control (physiological saline: PS) are indicated by * $p \leq 0.05$.

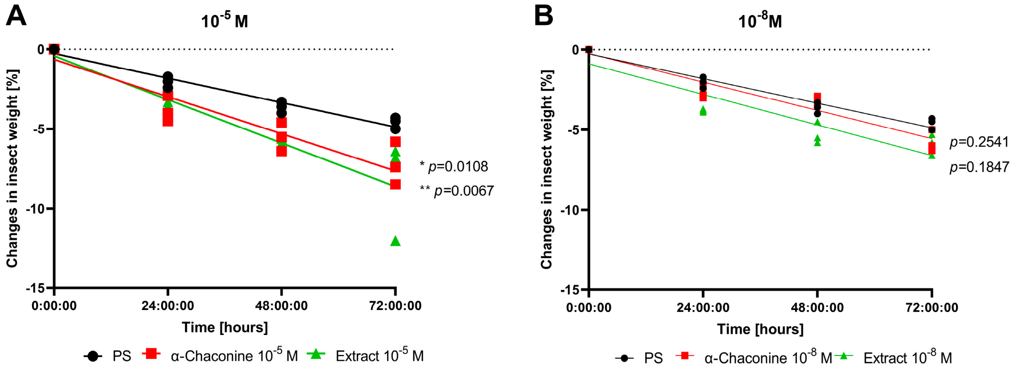

Figure S2. Scatter dot plots of graphs presented in Figure 2 presenting weight loss of *T. molitor* over 72 hours' time after application of α-chaconine (red) and *S. tuberosum* extract (green) at concentrations of 10^{-5} (A) and 10^{-8} M (B). Values are presented as mean ± SEM for N=3, n=60. Linear regression analysis was used to assess differences among the curves. Significant difference from the control (physiological saline: PS) are indicated by * $p \leq 0.05$ or ** $p \leq 0.01$.

	Concentration (M)	Gut motility (%)	Weight Loss (%)
**Control**	0	0	-4,6
**Chaconine**	10^{-5}	53,47	-7,2333
**Extract**	10^{-5}	49,05	-8,4
**Chaconine**	10^{-8}	54,29	-5,7667
**Extract**	10^{-8}	14,09	-5,9

Figure S3. Raw data used to generate correlation matrix presented in Figure 3. Values of percentage changes in gut motility and weight loss present changes observed at specific concentrations, control “concentration” was set to 0.

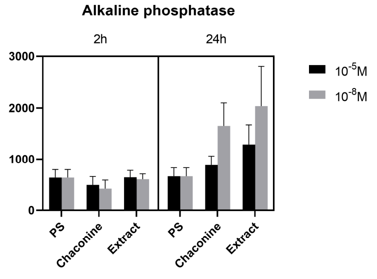

Tukey's multiple comparisons test	Summary	Adjusted P Value
PS:10^{-5}M 24h vs. Extract:10^{-8}M 24h	*	0,0349
PS:10^{-8}M 24h vs. Extract:10^{-8}M 24h	*	0,0349
Chaconine:10^{-5}M 2h vs. Extract:10^{-8}M 24h	*	0,0444
Chaconine:10^{-8}M 2h vs. Extract:10^{-8}M 24h	*	0,0295

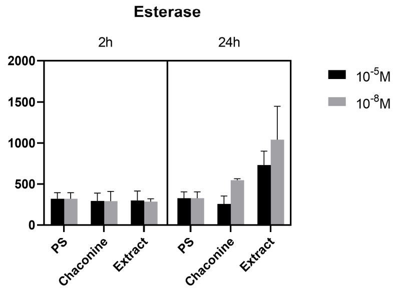

Tukey's multiple comparisons test	Summary	Adjusted P Value
PS:10^{-5}M 2h vs. Extract:10^{-8}M 24h	**	0,0095
PS:10^{-8}M 2h vs. Extract:10^{-8}M 24h	**	0,0095
PS:10^{-5}M 24h vs. Extract:10^{-8}M 24h	*	0,0103
PS:10^{-8}M 24h vs. Extract:10^{-8}M 24h	*	0,0103
Chaconine:10^{-5}M 2h vs. Extract:10^{-8}M 24h	*	0,0267
Chaconine:10^{-8}M 2h vs. Extract:10^{-8}M 24h	*	0,0255
Chaconine:10^{-5}M 24h vs. Extract:10^{-8}M 24h	*	0,0166
Extract:10^{-5}M 2h vs. Extract:10^{-8}M 24h	*	0,0288
Extract:10^{-8}M 2h vs. Extract:10^{-8}M 24h	*	0,0239

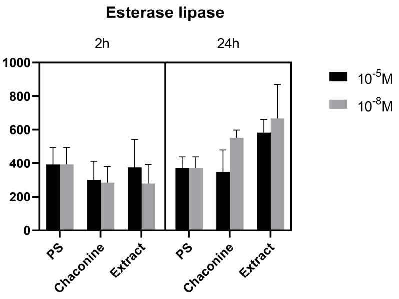

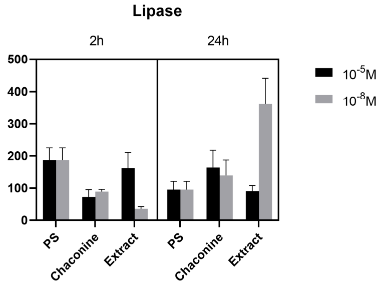

Tukey's multiple comparisons test	Summary	Adjusted P Value
PS:10^{-5}M 24h vs. Extract:10^{-8}M 24h	**	0,0019
PS:10^{-8}M 24h vs. Extract:10^{-8}M 24h	**	0,0019
Chaconine:10^{-5}M 2h vs. Extract:10^{-8}M 24h	**	0,0028
Chaconine:10^{-8}M 2h vs. Extract:10^{-8}M 24h	**	0,0058
Chaconine:10^{-8}M 24h vs. Extract:10^{-8}M 24h	*	0,0451
Extract:10^{-8}M 2h vs. Extract:10^{-8}M 24h	***	0,0005
Extract:10^{-5}M 24h vs. Extract:10^{-8}M 24h	***	0,0002

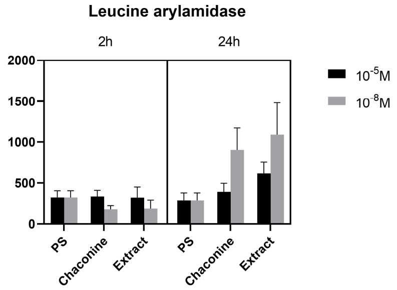

Tukey's multiple comparisons test	Summary	Adjusted P Value
PS:10^{-5}M 2h vs. Extract:10^{-8}M 24h	*	0,018
PS:10^{-8}M 2h vs. Extract:10^{-8}M 24h	*	0,018
PS:10^{-5}M 24h vs. Extract:10^{-8}M 24h	**	0,008
PS:10^{-8}M 24h vs. Extract:10^{-8}M 24h	**	0,008
Chaconine:10^{-8}M 2h vs. Extract:10^{-8}M 24h	**	0,0099
Extract:10^{-5}M 2h vs. Extract:10^{-8}M 24h	*	0,0499
Extract:10^{-8}M 2h vs. Extract:10^{-8}M 24h	*	0,011

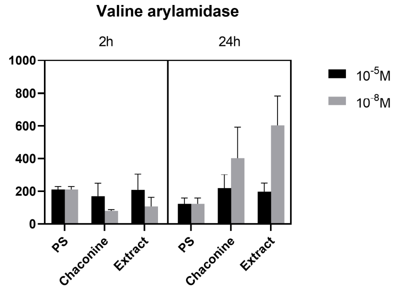

Tukey's multiple comparisons test	Summary	Adjusted P Value
PS:10^{-5}M 2h vs. Extract:10^{-8}M 24h	*	0,0226
PS:10^{-8}M 2h vs. Extract:10^{-8}M 24h	*	0,0226
PS:10^{-5}M 24h vs. Extract:10^{-8}M 24h	*	0,0131
PS:10^{-8}M 24h vs. Extract:10^{-8}M 24h	*	0,0131
Chaconine:10^{-5}M 2h vs. Extract:10^{-8}M 24h	*	0,0341
Chaconine:10^{-8}M 2h vs. Extract:10^{-8}M 24h	**	0,0052
Extract:10^{-8}M 2h vs. Extract:10^{-8}M 24h	**	0,0093
Extract:10^{-5}M 24h vs. Extract:10^{-8}M 24h	**	0,0018

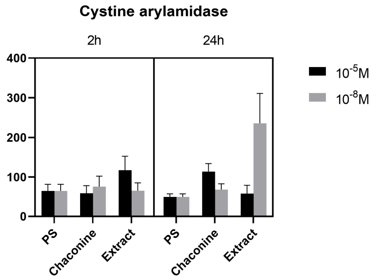

Tukey's multiple comparisons test	Summary	Adjusted P Value
PS:10^{-5}M 2h vs. Extract:10^{-8}M 24h	**	0,0087
PS:10^{-8}M 2h vs. Extract:10^{-8}M 24h	**	0,0087
PS:10^{-5}M 24h vs. Extract:10^{-8}M 24h	**	0,0015
PS:10^{-8}M 24h vs. Extract:10^{-8}M 24h	**	0,0015
Chaconine:10^{-5}M 2h vs. Extract:10^{-8}M 24h	**	0,006
Chaconine:10^{-8}M 2h vs. Extract:10^{-8}M 24h	*	0,0169
Chaconine:10^{-8}M 24h vs. Extract:10^{-8}M 24h	*	0,0106
Extract:10^{-8}M 2h vs. Extract:10^{-8}M 24h	**	0,009
Extract:10^{-5}M 24h vs. Extract:10^{-8}M 24h	**	0,0054

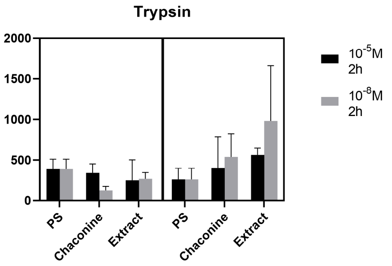

Tukey's multiple comparisons test	Summary	Adjusted P Value
PS:10^{-5}M 2h vs. Extract:10^{-8}M 24h	*	0,0336
PS:10^{-8}M 2h vs. Extract:10^{-8}M 24h	*	0,0336
PS:10^{-5}M 24h vs. Extract:10^{-8}M 24h	**	0,004
PS:10^{-8}M 24h vs. Extract:10^{-8}M 24h	**	0,004
Chaconine:10^{-8}M 2h vs. Extract:10^{-8}M 24h	**	0,0027
Extract:10^{-5}M 2h vs. Extract:10^{-8}M 24h	*	0,0171
Extract:10^{-8}M 2h vs. Extract:10^{-8}M 24h	*	0,0225

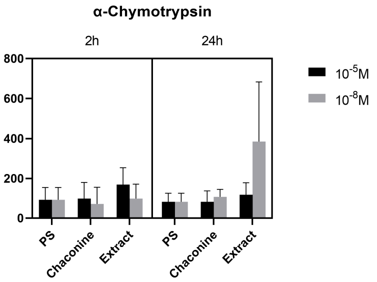

Tukey's multiple comparisons test	Summary	Adjusted P Value
PS:10^{-5}M 2h vs. Extract:10^{-8}M 24h	*	0,0192
PS:10^{-8}M 2h vs. Extract:10^{-8}M 24h	*	0,0192
PS:10^{-5}M 24h vs. Extract:10^{-8}M 24h	*	0,0391
PS:10^{-8}M 24h vs. Extract:10^{-8}M 24h	*	0,0391
Chaconine:10^{-8}M 2h vs. Extract:10^{-8}M 24h	*	0,0286
Chaconine:10^{-5}M 24h vs. Extract:10^{-8}M 24h	*	0,0392

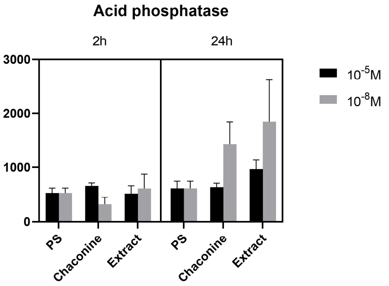

Tukey's multiple comparisons test	Summary	Adjusted P Value
PS:10^{-5}M 2h vs. Extract:10^{-8}M 24h	*	0,0152
PS:10^{-8}M 2h vs. Extract:10^{-8}M 24h	*	0,0152
PS:10^{-5}M 24h vs. Extract:10^{-8}M 24h	*	0,0214
PS:10^{-8}M 24h vs. Extract:10^{-8}M 24h	*	0,0214
Chaconine:10^{-8}M 2h vs. Extract:10^{-8}M 24h	*	0,0102
Extract:10^{-5}M 2h vs. Extract:10^{-8}M 24h	*	0,04

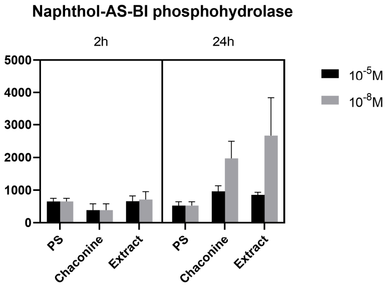

Tukey's multiple comparisons test	Summary	Adjusted P Value
PS:10^{-5}M 2h vs. Extract:10^{-8}M 24h	**	0,0018
PS:10^{-8}M 2h vs. Extract:10^{-8}M 24h	**	0,0018
PS:10^{-5}M 24h vs. Extract:10^{-8}M 24h	**	0,0012
PS:10^{-8}M 24h vs. Extract:10^{-8}M 24h	**	0,0012
Chaconine:10^{-5}M 2h vs. Extract:10^{-8}M 24h	**	0,0024
Chaconine:10^{-8}M 2h vs. Extract:10^{-8}M 24h	**	0,0024
Extract:10^{-5}M 2h vs. Extract:10^{-8}M 24h	*	0,011
Extract:10^{-8}M 2h vs. Extract:10^{-8}M 24h	*	0,0144
Extract:10^{-5}M 24h vs. Extract:10^{-8}M 24h	*	0,029

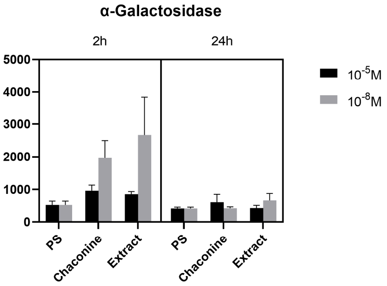

Tukey's multiple comparisons test	Summary	Adjusted P Value
PS:10^{-5}M 2h vs. Extract:10^{-8}M 2h	**	0,0014
PS:10^{-8}M 2h vs. Extract:10^{-8}M 2h	**	0,0014
PS:10^{-5}M 24h vs. Chaconine:10^{-8}M 2h	*	0,0475
PS:10^{-5}M 24h vs. Extract:10^{-8}M 2h	***	0,0007
PS:10^{-8}M 24h vs. Chaconine:10^{-8}M 2h	*	0,0475
PS:10^{-8}M 24h vs. Extract:10^{-8}M 2h	***	0,0007
Chaconine:10^{-5}M 24h vs. Extract:10^{-8}M 2h	**	0,009
Chaconine:10^{-8}M 24h vs. Extract:10^{-8}M 2h	**	0,0033
Extract:10^{-8}M 2h vs. Extract:10^{-5}M 24h	**	0,0034
Extract:10^{-8}M 2h vs. Extract:10^{-8}M 24h	*	0,012

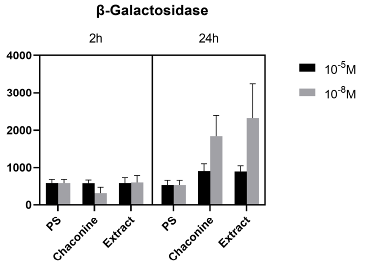

Tukey's multiple comparisons test	Summary	Adjusted P Value
PS:10^{-5}M 2h vs. Extract:10^{-8}M 24h	**	0,0013
PS:10^{-8}M 2h vs. Extract:10^{-8}M 24h	**	0,0013
PS:10^{-5}M 24h vs. Chaconine:10^{-8}M 24h	*	0,0365
PS:10^{-5}M 24h vs. Extract:10^{-8}M 24h	***	0,0008
PS:10^{-8}M 24h vs. Chaconine:10^{-8}M 24h	*	0,0365
PS:10^{-8}M 24h vs. Extract:10^{-8}M 24h	***	0,0008
Chaconine:10^{-5}M 2h vs. Extract:10^{-8}M 24h	**	0,008
Chaconine:10^{-8}M 2h vs. Chaconine:10^{-8}M 24h	*	0,0344
Chaconine:10^{-8}M 2h vs. Extract:10^{-8}M 24h	**	0,0013
Extract:10^{-5}M 2h vs. Extract:10^{-8}M 24h	**	0,0082
Extract:10^{-8}M 2h vs. Extract:10^{-8}M 24h	**	0,009
Extract:10^{-5}M 24h vs. Extract:10^{-8}M 24h	*	0,0345

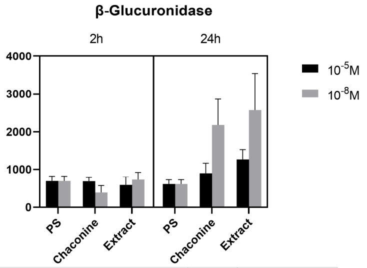

Tukey's multiple comparisons test	Summary	Adjusted P Value
PS:10^{-5}M 2h vs. Chaconine:10^{-8}M 24h	*	0,0312
PS:10^{-5}M 2h vs. Extract:10^{-8}M 24h	**	0,0023
PS:10^{-8}M 2h vs. Chaconine:10^{-8}M 24h	*	0,0312
PS:10^{-8}M 2h vs. Extract:10^{-8}M 24h	**	0,0023
PS:10^{-5}M 24h vs. Chaconine:10^{-8}M 24h	*	0,0185
PS:10^{-5}M 24h vs. Extract:10^{-8}M 24h	**	0,0013
PS:10^{-8}M 24h vs. Chaconine:10^{-8}M 24h	*	0,0185
PS:10^{-8}M 24h vs. Extract:10^{-8}M 24h	**	0,0013
Chaconine:10^{-5}M 2h vs. Extract:10^{-8}M 24h	*	0,0123
Chaconine:10^{-8}M 2h vs. Chaconine:10^{-8}M 24h	*	0,0208
Chaconine:10^{-8}M 2h vs. Extract:10^{-8}M 24h	**	0,0021
Chaconine:10^{-5}M 24h vs. Extract:10^{-8}M 24h	*	0,0406
Extract:10^{-5}M 2h vs. Extract:10^{-8}M 24h	**	0,007
Extract:10^{-8}M 2h vs. Extract:10^{-8}M 24h	*	0,0158

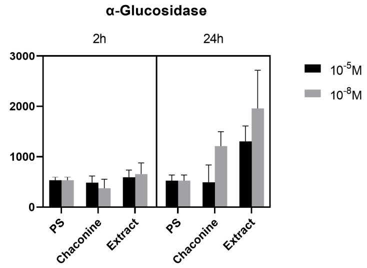

Tukey's multiple comparisons test	Summary	Adjusted P Value
PS:10^{-5}M 2h vs. Extract:10^{-8}M 24h	**	0,003
PS:10^{-8}M 2h vs. Extract:10^{-8}M 24h	**	0,003
PS:10^{-5}M 24h vs. Extract:10^{-8}M 24h	**	0,0028
PS:10^{-8}M 24h vs. Extract:10^{-8}M 24h	**	0,0028
Chaconine:10^{-5}M 2h vs. Extract:10^{-8}M 24h	*	0,0115
Chaconine:10^{-8}M 2h vs. Extract:10^{-8}M 24h	**	0,005
Chaconine:10^{-5}M 24h vs. Extract:10^{-8}M 24h	*	0,0121
Extract:10^{-5}M 2h vs. Extract:10^{-8}M 24h	*	0,0261
Extract:10^{-8}M 2h vs. Extract:10^{-8}M 24h	*	0,0407

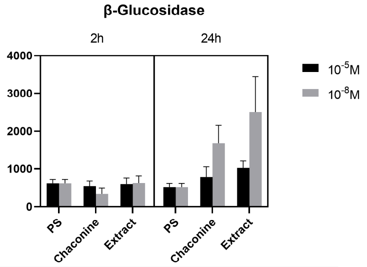

Tukey's multiple comparisons test	Summary	Adjusted P Value
PS:10^{-5}M 2h vs. Extract:10^{-8}M 24h	***	0,0003
PS:10^{-8}M 2h vs. Extract:10^{-8}M 24h	***	0,0003
PS:10^{-5}M 24h vs. Extract:10^{-8}M 24h	***	0,0001
PS:10^{-8}M 24h vs. Extract:10^{-8}M 24h	***	0,0001
Chaconine:10^{-5}M 2h vs. Extract:10^{-8}M 24h	**	0,0016
Chaconine:10^{-8}M 2h vs. Extract:10^{-8}M 24h	***	0,0004
Chaconine:10^{-5}M 24h vs. Extract:10^{-8}M 24h	**	0,0088
Extract:10^{-5}M 2h vs. Extract:10^{-8}M 24h	**	0,0024
Extract:10^{-8}M 2h vs. Extract:10^{-8}M 24h	**	0,0028
Extract:10^{-5}M 24h vs. Extract:10^{-8}M 24h	*	0,0241

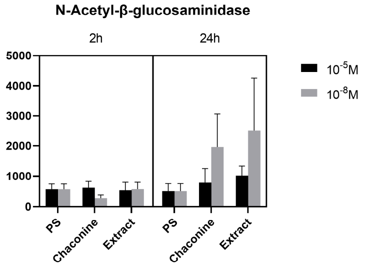

Tukey's multiple comparisons test	Summary	Adjusted P Value
PS:10^{-5}M 2h vs. Extract:10^{-8}M 24h	**	0,0014
PS:10^{-8}M 2h vs. Extract:10^{-8}M 24h	**	0,0014
PS:10^{-5}M 24h vs. Chaconine:10^{-8}M 24h	*	0,0274
PS:10^{-5}M 24h vs. Extract:10^{-8}M 24h	***	0,0005
PS:10^{-8}M 24h vs. Chaconine:10^{-8}M 24h	*	0,0274
PS:10^{-8}M 24h vs. Extract:10^{-8}M 24h	***	0,0005
Chaconine:10^{-5}M 2h vs. Extract:10^{-8}M 24h	**	0,0077
Chaconine:10^{-8}M 2h vs. Chaconine:10^{-8}M 24h	*	0,0268
Chaconine:10^{-8}M 2h vs. Extract:10^{-8}M 24h	***	0,0009
Chaconine:10^{-5}M 24h vs. Extract:10^{-8}M 24h	*	0,0203
Extract:10^{-5}M 2h vs. Extract:10^{-8}M 24h	**	0,0047
Extract:10^{-8}M 2h vs. Extract:10^{-8}M 24h	**	0,0059

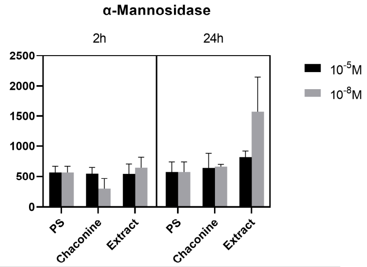

Tukey's multiple comparisons test	Summary	Adjusted P Value
PS:10^{-5}M 2h vs. Extract:10^{-8}M 24h	*	0,0268
PS:10^{-8}M 2h vs. Extract:10^{-8}M 24h	*	0,0268
PS:10^{-5}M 24h vs. Extract:10^{-8}M 24h	*	0,0291
PS:10^{-8}M 24h vs. Extract:10^{-8}M 24h	*	0,0291
Chaconine:10^{-8}M 2h vs. Extract:10^{-8}M 24h	**	0,01
Extract:10^{-5}M 24h vs. Extract:10^{-8}M 24h	*	0,0231

Figure S4. Changes in digestive enzyme activity at 2 h and 24 h after injection of α-chaconine, S. tuberosum extract, or physiological saline (PS) at concentrations of 10^{-5} M and 10^{-8} M. Statistical significances between groups are presented in the tables below the graphs; groups that did not differ significantly (ns) are not included in the tables. Enzyme activity data were analyzed using a three-way analysis of variance (ANOVA) with treatment (PS, α-chaconine, S. tuberosum extract), dose (10^{-5} M and 10^{-8} M), and time (2 h and 24 h) as fixed factors. All data are expressed as mean ± SD. Differences were considered statistically significant at the following levels: $p \leq 0.05$ (*), $p \leq 0.01$ (**), $p \leq 0.001$ (***) or $p \leq 0.0001$ (****).
